# Efficient Removal of Dissolved Organics and Naphthenic Acids from Offshore Produced Water: Performance and Challenges of an Electrochemical Pilot Plant

**DOI:** 10.1021/acsomega.6c02682

**Published:** 2026-08-03

**Authors:** Daniela Gier Della Rocca, Silvio Edegar Weschenfelder, Marta Cordeiro da Fonseca, Glaucia Rodrigues Vieira de Almeida da Fonseca, Luciana Prazeres Mazur, Sálvio Lima de Carvalho Neto, Adriano da Silva, Débora de Oliveira, Regina F. P. M. Moreira

**Affiliations:** † Laboratory of Energy and Environment − LEMA, Department of Chemical and Food Engineering, Federal University of Santa Catarina, Florianópolis 88040-970, Brazil; ‡ Petrobras Research Center − CENPES, 21941-915 Rio de Janeiro, RJ, Brazil; § Mass Transfer Laboratory − LABMASSA, Department of Chemical and Food Engineering, Federal University of Santa Catarina, Florianópolis 88040-970, Brazil

## Abstract

Oilfield produced water (OPW) is a complex wastewater, including recalcitrant compounds and stable emulsions. Although electrochemical treatment has shown promising laboratory-scale results for treating this effluent, pilot-scale validation and scale-up remain poorly explored. Therefore, this study investigates the treatment of real offshore OPW using two electrochemical pilot plants (6 and 15 L). Under the optimum operating conditions (25 A·m^–2^ and a residence time of 4 min), the process reduced total oil and grease from 152 to 3 mg·L^–1^, turbidity from 69.4 to 1.0 NTU, and total organic carbon from 561 to 42 mg·L^–1^. In addition, the larger pilot unit achieved similar or improved performance, confirming the feasibility of process scale-up. The results indicate that electrocoagulation/flocculation and electro-oxidation occurred simultaneously, promoting the rapid removal of dissolved organic compounds and anodic transformation of the naphthenic acids. Practical aspects relevant to offshore implementation, including energy demand (7.8 kWh kg of TOG removed^–1^), heat generation (166 W), gas evolution (13.5 L·h^–1^), and process footprint (10 m·h^–1^ and 15 h^–1^), are also discussed, highlighting the feasibility of industrial implementation.

## Graphical abstract

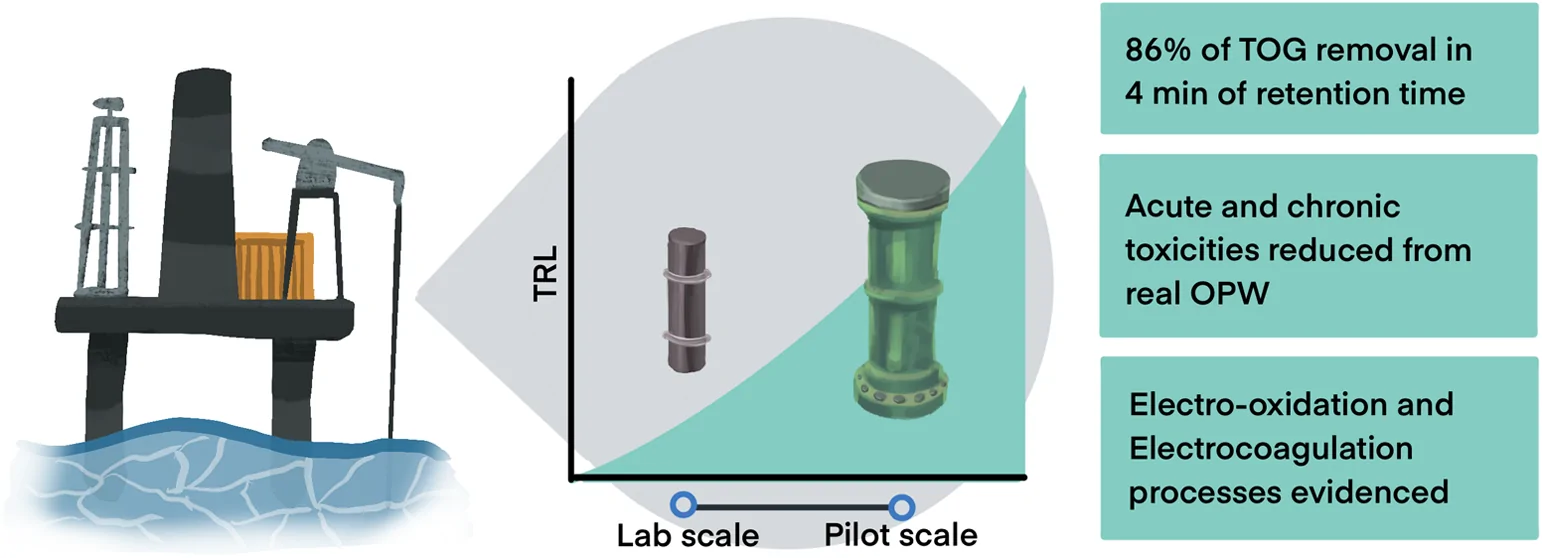

## 1 Introduction

Oilfield produced water (OPW) is a residue generated by the oil and gas (O&G) extraction and production process, resulting from the combination of formation water with injection water. Due to the high demand for petroleum and its derivatives, OPW is generated in large volumes and has a complex composition. It contains high concentrations of salts, inorganic ions, metals, radioactive materials, and several classes of dispersed and dissolved organic compounds (in particular aromatic molecules, which consistently contribute to natural environments’ toxicity), which contribute to the total oil and grease (TOG) content.^1^

Given these intricate characteristics, OPW treatment requires advanced processes, often involving a combination of separation technologies to enable either appropriate disposal or reuse.^1,2^ Since OPW composition varies considerably between extraction wells, conventional treatment methods, especially offshore, may struggle to effectively remove stable emulsions, fine oil droplets, and suspended oil and/or hazardous dissolved organic and inorganic components.^2^ This challenge is so critical that, in some cases, the O&G extraction rate is constrained by the large volume of OPW that must be appropriately treated for disposal or reuse, leading to economic losses and operational difficulties.^1^

Moreover, the complexity of offshore OPW treatment is increased by the weight, footprint, and energy demand constraints of the facilities, which impact capital costs and, consequently, the economic viability of greenfield projects and, to a greater extent, the feasibility of brownfield improvements. In this context, electrochemical techniques such as electrocoagulation (EC) and electro-oxidation (EO) have emerged in the literature as promising alternatives for OPW treatment.^3^ EC operates through a mechanical separation mechanism, where a sacrificial anode dissolves into the aqueous phase, releasing metal hydroxides (primarily Fe­(OH)_3_ or Al­(OH)_3_) that induce pollutant coagulation, leading to the formation of a metal–organic sludge.^4,5^ In contrast, EO relies on the generation of reactive oxygen species (ROS) through direct or indirect anodic oxidation, producing highly reactive radicals that degrade organic matter.^6^

In fact, some studies coupled EO and EC (either sequential or simultaneous) to treat real petroleum wastewaters with very promising results, with chemical oxygen demand (COD) removals reaching up to ≥ 92%.^6–9^ EC is an efficient and fast technique to remove suspended solids,^10,11^ while EO excels in oxidizing recalcitrant organic contaminants.^8^ Therefore, EC contributes to reducing film layer formation on the cathodes, as well as lower energy consumption. At the same time, EO operation reduces the sludge formation (nonsacrificial electrodes), the compounds can potentially achieve mineralization, and the effluent might be disinfected, consequent to the subproduct formation.^8,9^

Recently, Salman and Abbar (2023)^9^ investigated a simultaneous EO + EC system to treat oilfield effluent, using Al (EC), stainless steel (EO), and graphite (EO) electrodes. The authors observed a synergistic effect of the combined processes, which led to high COD removal (93%) with low energy consumption (128.6 kWh·kg COD^–1^).

Moreover, both processes have demonstrated high removal rates on the bench scale, showcasing excellent efficiency with low costs, simple operation, and a compact footprint.^3^ These advantages are crucial for real-world applications of oil platforms.

However, the current literature is still dominated by bench-scale studies using synthetic or simplified wastewater matrices,^2,12–14^ while pilot-scale validation and scale-up studies remain limited. In this sense, this study not only validates real offshore OPW treatment by electrochemical processes in a pilot-scale unit but also provides a new understanding of the reaction mechanisms and engineering analysis for offshore deployment. This research investigated the TOG removal efficiency in two electrochemical pilot units of different capacities (6 and 15 L) to advance the technology readiness level (TRL) from 5 to 6.^15^ The treatment efficiency, acute and chronic toxicity, process scale-up, and key factors for industrial-scale implementation, such as energy demand, heat generation, gas evolution, and footprint, using real offshore OPW under representative conditions of practical operation, are also discussed. Moreover, the study provides experimental evidence supporting the simultaneous occurrence of EC/flocculation and EO, promoting both rapid removal of dissolved organic compounds and anodic partial oxidation of NA, despite the challenges of working with a high-salinity wastewater.

## 2 Materials and Methods

### 2.1 Chemicals and Materials

A COD kit for high chlorine levels was purchased by Hach. Mercury sulfate (HgSO_4_, 98% purity) and hydrochloric acid were supplied by Êxodo Científica (37%, analytical purity). *n*-hexane EMSURE (99% purity) was acquired by Merck Ltd.a. *Aliivibrio fischeri* bacteria and *Echinoidea* sea urchin were provided by CENPES analytical laboratory. All reagents necessary for the analyses were used as supplied without further purification. All purities were at least analytical grades.

### 2.2 Produced Water Sampling and Characterization

A total of 1000 L of real offshore OPW was collected at an oilfield platform located on the southeast Brazilian Coast in January 2024. The sample was stored at room temperature in an intermediate bulk container (IBC) made of high-density polyethylene until the electrochemical tests on a pilot scale were conducted in April 2024. Since OPW properties might vary over time (phase separation, biodegradation, and/or volatilization), all samples were analyzed when sent back to Brazil. However, TOG was analyzed before, during, and after the experiments’ execution. The results showed low variability (±5 mg·L^–1^), indicating that it remained stable over time. The OPW (sample S0) characteristics are presented in Table 1. The analytical procedures (Table 1) are described in detail in Section 3.4. It is important to highlight that the absence of the total petroleum hydrocarbons (TPH, dispersed oil), combined with elevated TOG and COD, denotes that the O&G content is predominantly composed of dissolved oils.

**Table 1:** Physicochemical Properties of the Real Brazilian Offshore OPW Sample

analytical parameter	value
pH	7.18
conductivity (mS·cm^–1^)	59.09
salinity (g·L^–1^)	34
turbidity (NTU)	69.4
chemical oxygen demand (COD, O_2_ mg·L^–1^)	1800
O&G content (mg·L^–1^)	152
total petroleum hydrocarbons (TPH, mg·L^–1^)	0

### 2.3 Electrochemical Pilot-Scale Experiments

The tests were conducted in two pilot plants of different sizes; their details are presented in Table 2.

**Table 2:** Reactor Characteristics and Operation Conditions Applied to the Electrochemical Treatment of Real Offshore OPW in the Pilot Plant Units

parameter	unit 1	unit 2
volume (L)	6	15
flow rate (L·h^–1^)	45–180	220
flow orientation^16,17^	tangential	tangential
electrodes (mol %)^16,17^	Al (100) or Ti/MMO (PtO_2_, 0–10; IrO_2_, 10–30; RuO_2_, 10–40; and Ta_2_O_5_, 10–35)	Al (100) or Ti/MMO (PtO_2_, 0–10; IrO_2_, 10–30; RuO_2_, 10–40; and Ta_2_O_5_, 10–35)
voltage (V)	3–6	18–22
current density (A·m^–2^)	25	25
geometrical area of the cells (cm^2^)	595	5200

Both systems were composed by a feeding tank, an electrochemical reactor, a strainer (a 20 L separator vessel with an inlet at top side and three outlets: one at the bottom for solids, one at the downside for treated aqueous wastewater, and one at the top side for gas and/or oil), and a filtration system with a 50 μm cutoff, as shown in the scheme in Figure 1. Samples were collected at 4 points (Figure 1): (A) at the electrochemical reactor inlet, (B) at the electrochemical reactor outlet, (C) at the strainer outlet, and (D) at the filter outlet.

**Figure 1:**
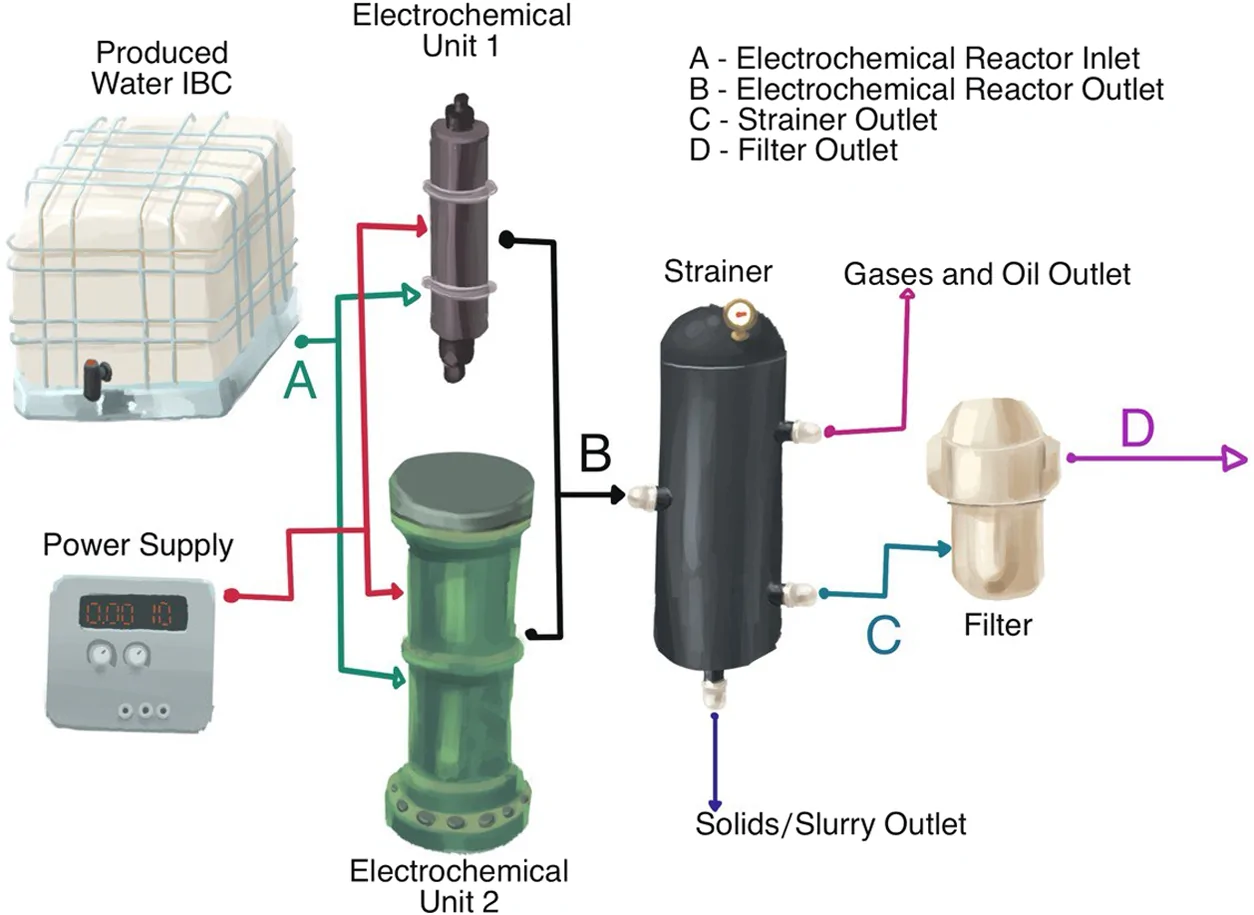
Schematic setup applied to the electrochemical treatment containing a feeding tank, a power supply, an electrochemical reactor (Unit 1 or 2), a strainer, and a filter outlet pilot.

The tests on Unit 1 were carried out to evaluate the effect of residence time (RT) on TOG removal. Experiments were performed at 2 (sample S1), 4 (sample S2), and 8 (sample S3) min of RT. Current density (CD) was established as 25 A·m^–2^. In addition, a blank test with filtration only (sample S4), i.e., the electric system was shut off, was performed. The residence time that resulted in the highest TOG removal efficiency on Unit 1 was selected to evaluate the scale-up condition in Unit 2 (sample S5).

After allowing sufficient time for the system to achieve a steady state for each condition (constant voltage and hydraulic flow), samples were collected at the sampling points A–D for characterization (Figure 1). For toxicity analyses, samples were collected in plastic containers and immediately frozen, while the sampling flasks for TOG analyses were stored in amber glass bottles and maintained at room temperature. An additional 20 L sampling was acquired from the best evaluated condition on unit 1 and stored in a high-density polyethylene drum at room temperature, to characterize it more deeply.

It is important to point out that replicate tests, as well as tests in other CDs and pHs, were not performed due to the limited sample volume available during pilot-scale operation. The feeding volume was insufficient to conduct the experiments without affecting the process stability. However, it is important to highlight that in order to guarantee the representativeness of the collected samples, their collection was conducted after the system had reached steady-state operation (±20 min) and collected for at least three RT afterwords, to ensure the fidelity characteristics of the samples, which is commonly considered sufficient for complete renewal of the reactor contents under continuous-flow condition.^18^

Furthermore, as the primary objective was to assess process performance, hydraulic behavior, and scale-up feasibility under realistic pilot conditions, a single CD condition and natural pH were selected to ensure stable operation and reliable comparison between the two electrochemical units. The current density was fixed at 25 A·m^–2^. This value was chosen within the low range of typically reported CDs for produced water and petroleum wastewater treatment,^19^ but it also reported high removal efficiency.^20–23^ This condition was selected to provide sufficient electrochemical activity for coagulant generation, efficient gas-assisted flotation, and sufficient partial oxidation (enough to reduce TOG), while minimizing excessive energy demand, heat generation, electrode abrasion, and chlorine subproducts formation.^19^ Similarly, although pH strongly affects EC and EO processes, pH adjustment was intentionally avoided. From a real-life offshore implementation perspective, acidification and/or alkalinization would substantially increase chemical consumption, corrosion impacts, safety risks, sludge generation, and operational and logistics complexities. Therefore, experiments were conducted at the natural pH of the real OPW to better represent practical operating conditions.

In addition, a conventional chemical treatment on an oilfield platform offshore was conducted to compare the results. Briefly, 1.5 L of the same OPW sample was acidified to pH 5, then 11 mL of a commercial flocculant (carbamide, 30 mg·L^–1^) was added to the solution, and air bubbles were injected to conduct the flotation process in a flotation column with a porous plate at room temperature. Air (4 bar) was used as the carrier gas. The specified parameters for this experiment were based on real offshore platform treatment conditions.

### 2.4 Analytical Analyses

TOG content, acute toxicity, chronic toxicity, and solids (total, fixed, and volatile) were determined in accordance with SM 5520-B, ISO 11348-3, ABNT NBR 15350, and ISO 2540-G^24–27^ protocols, respectively. TPH was determined similarly to O&G content, but the pH was adjusted to 7 instead of 2 before the extraction step. Without acidification, the mass transfer of soluble molecules is inhibited because lower pH reduces the solubility of NA, causing it to migrate to the organic phase. Thereby, the dispersed oil fraction (TPH) can be determined. COD was measured using a kit for high chlorine levels (HR Plus, Hach) with the addition of 0.5 g of HgSO_4_, as indicated by the supplier, for Cl concentrations of up to 40 g·L^–1^. Moreover, ζ potential and superficial tension of some samples were measured by a dynamic light scattering (DLS) analyzer (Anton-Paar, model Litesizer DLS 500) and a goniometer (Ramé-hart, tensiometer Model 250). Respectively, pH, conductivity, and salinity were, in this order, also measured by a pH meter (Marte Científica, model MC35), a conductivity meter (Digimed, model DM-32), and a refractometer (Lorben, model GT809).

Furthermore, the initial sample (S0), the strainer output with a RT of 4 min (S2–C), and the chemical treatment samples underwent complete characterization following well-established protocols^28–33^ in accordance with Brazil’s legislation.^34,35^

At last, the extracts of S0 and S2-C after the TOG analysis were solubilized in *n*-hexane and analyzed by gas chromatography–mass spectrometry (GC/MS). The analyses were performed on the Agilent GC 7890A equipped with the Agilent 5975C mass detector (MS). The capillary column used was an HP-5MS (Agilent), made of fused silica (length of 30 m × internal diameter of 250 μm × film thickness of 0.25 μm, composed of 5% phenyl-95% methylpolysiloxane), and connected to an electron impact ionization (EI) source operating at 70 eV with a quadrupole mass analyzer. The mass scan was from 42 to 600 m·z^–1^ in positive mode. Helium 5.0 was used as a carrier gas at a constant flow of 1.0 mL·min^–1^. The temperatures of the injector (operating in splitless mode) and the interface were 330 and 300 °C, respectively. The solvent delay was 3.0 min. The injection volume was 0.3 μL with the Agilent GC Sampler 80 autosampler equipped with a 10 μL syringe. The oven temperature program consisted of 100 °C for 5 min, followed by a temperature increase of 100 °C (5 °C·min^–1^) to 325 °C for 10 min. The total analysis execution time was 60 min.

### 2.5 Operational Parameters Evaluation

To compare the performance of the electrochemical plants at different scales (units 1 and 2), the collected data were used to evaluate key points for larger-scale applications and to identify scale-up limitations.

The CD (A·m^–2^), electrical charge (J, A·h^–1^m^–3^), electrical power (W, kW), electrical energy costs (EEC, USD), and specific energy consumption (*E*_C_, kWh·kg of TOG removed^–1^) were calculated as below (eqs 1–5). The average cost of electrical energy production (0.11 USD·kWh^–1^) took into consideration the value for its generation in floating offshore platforms by gas combustion in turbines.^36^

I $$\mathrm{CD}=\frac{i}{{1000A}_{\mathrm{cell}}}$$

II $$J=\frac{i}{Q}$$

III $$W=\frac{\mathrm{iU}}{1000}$$

IV $$\mathrm{EEC}=\frac{W\times 0.11}{1000Q}$$

V $$E_{C}=\frac{1000\mathrm{Ui}}{Q\left({\mathrm{TOG}}_{f}-{\mathrm{TOG}}_{0}\right)}$$

where *i* is the electrical current (*A*), *A*_cell_ is the total geometric area of the cells, *Q* is the volumetric flow rate (m^3^·h^–1^), *U* is the applied voltage (V), and TOG_f_ and TOG_0_ are the final and initial O&G concentrations (mg·L^–1^), respectively.

The H_2_, O_2_, and Cl_2_ volumetric flows estimation was calculated as in eq 6:^37^

VI $$g\mathrm{as generation}=\frac{i}{\mathrm{nF}}$$

where *n* is the number of electrons transferred, and *F* is the Faraday′s constant (96,500 C·mol^–1^).

The heat generation (*q*, kW) was calculated using practical energy efficiency (ε cell) values already reported in the literature (eq 7),^38^ which accounted for charge transfers and the conversion of ohmic losses into heat. Moreover, the water evaporation (*m*_W_) was calculated considering the sensitive heat equation (eq 8).

VII $$q=\left(1-\varepsilon_{\mathrm{cell}}\right)\cdot W$$

VIII $$m_{w}=\frac{q}{c\Delta t}$$

where *c* is the specific heat of the water constant (2260 J·g^–1^), and Δ*t* is the time of operation (s).

Capacity by area (*F*_A_) and capacity by volume (*F*_V_) (eqs 9 and 10) were used to evaluate the required footprint of the offshore platform.^39^

IX $$F_{A}=\frac{Q}{A}$$

X $$F_{v}=\frac{Q}{V}$$

where *Q* is the mean flow rate, *A* is the projected floor area required by the equipment, and *V* is the equipment volume. High *F*_A_ and *F*_V_ values represent higher processing capacity of OPW under the same area or volume, i.e., reduced footprint and size needed for similar treatment.

## 3 Results and Discussion

### 3.1 Effect of the Operating Conditions on the Electrochemical Treatment Efficiency

First of all, filtration alone did not have a considerable impact on the treatment process, as it did not substantially alter the pH, conductivity, salinity, and O & G content parameters. The only factor notably affected by filtration was turbidity, which decreased as a consequence of the retention of larger particles by the filter, contributing to lower measures of this parameter.

The CD was initially set at 25 A·m^–2^, and the OPW flow rate was adjusted in order to establish a residence time of 4 min for tests using Unit 1. In addition to the reactions involving dissolved pollutants, the formation of gases (oxygen, hydrogen, and chlorine) was expected,^40^ promoting the evolution of these gases (eqs 11–13).

XI $$2H_{2}O\to H_{2}+2{\mathrm{OH}}^{-}$$

XII $$4{\mathrm{OH}}^{-}\to O_{2}+2H_{2}O+4e^{-}$$

XIII $$2{\mathrm{Cl}}^{-}\to {\mathrm{Cl}}_{2}+2e^{-}$$

Therefore, the reactor operated under slightly pressure (≈7 psig), and hydrogen, oxygen, and chlorine could be partially dissolved in the liquid phase. As the pressure on the strainer was reduced, the bubbles were released, promoting a flotation-like process. At the outlet of the electrochemical reactor (point B, Figure 1), a supernatant similar to dispersed oil was observed (Figure 2). This supernatant might be subproducts with lower polarity than the dissolved oils, such as naphthenic acids (NA). Literature reports decarboxylation as one of the most common oxidation mechanisms for NA.^41–43^ Therefore, if these molecules lose the carboxylic acid group, their polarity is reduced. Thus, it may cause another apolar phase to be formed, which could not be detected in the subsequent steps of the process, indicating that a gravitational separation occurred in the strainer.

**Figure 2:**
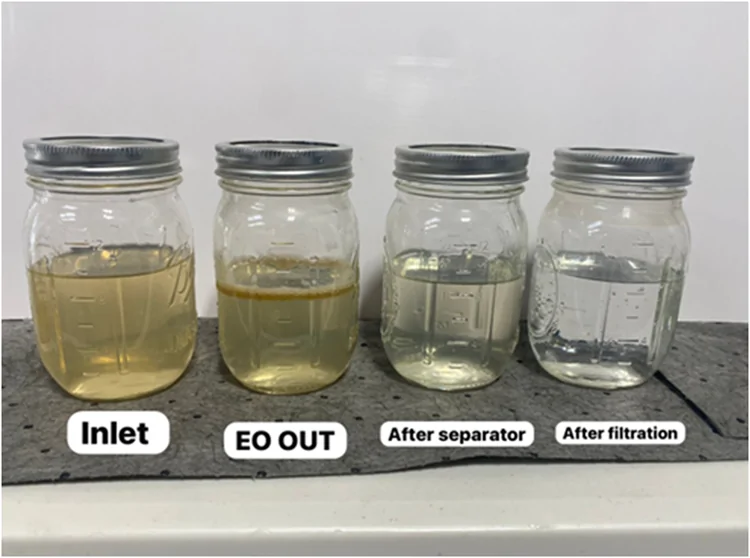
Collected samples at each step of the treatment process in Unit 1, applying 25 A·m^–2^ and 4 min of RT. From left to right: electrochemical reactor inlet (current A), electrochemical reactor outlet (current B), strainer outlet (current C), and filter outlet (current D) (Photograph taken by R F P M Moreira).

Furthermore, the turbidity (Table 3) decreases throughout the treatment, suggesting that the removal of oils, dissolved organics, and solids from the original wastewater sample was achieved.

**Table 3:** Samples Identification, Operational Conditions, and Analytic Analyses Results from Tests Conducted in Unit 1 at 25 A·m^–2^

sample identification^a^	sampling point	retention time (min)	pH	conductivity (mS·cm^–1^)	salinity (g·L^–1^)	turbidity (NTU)	TOG content (mg·L^–1^)
S0	A		7.18	59.09	34	69.4	152
S1-B	B	2	7.41	79.75		58.0	113
S1-C	C	2	8.15	26.40	12	9.4	30
S1-D	D	2	6.89	29.55	9	0.6	22
S2-B	B	4	6.26	52.53		29.0	127
S2-C	C	4	5.35	11.36	1	1.0	3
S2-D	D	4	6.71	18.92	9	0.9	2
S3-B	B	8	7.16	51.94		1.3	61
S3-C	C	8	7.64	27.60	9	1.3	24
S3-D	D	8	3.85	14.84	2	0.5	1
S4	D	4	6.65	60.62	34	6.4	141

a S0: raw OPW; S1, S2, and S3: treated OPW at 25 A·m^–2^ CD with RT 2, 4, or 8 min, respectively. S4: filtration only. Sampling points: A, B, C, and D: electrochemical reactor inlet, electrochemical reactor outlet, strainer outlet, and filter outlet, respectively.

In terms of physicochemical characterization, the results showed that, in general, samples increased their acidity throughout the process (Table 3). This fact suggests that radicals continued to react even after the strainer, since pH decreased, which might be associated with the oxidation reactions that kept occurring, and might lead to the formation of acidic subproducts, such as carboxylic acids.^44,45^

Moreover, conductivity, turbidity, and salinity have interconnected responses. As can be observed (Table 3), salinity drops drastically independently of the RT applied, reducing by at least 65% from the initial value. This is associated with the EO reactions that occur on the anodes and cathodes, transforming the NaCl from the offshore OPW into other subproducts. The chlorine evolution reactions lead to Cl^–^ oxidation, forming several chlorine species such as HClO, ClO_2_, Cl_2_O, and Cl_2_ (eqs 13–17).^44^ On the other hand, hydrogen evolution causes OH^–^ to be formed due to water hydrolysis (eq 18). Therefore, as Na^+^ has high electropositivity, it rapidly reacts with these anions, forming NaOH (eq 19).^44,46^ It is also relevant to notice that, as salinity was measured by refractometry, phase separation, emulsion breaking, and the complex composition of OPW might cause light scattering, beam deviation, and unstable refractive index readings, interfering with the light transmission through the aqueous medium. Thus, leading to considerable differences in the salinity for treated and untreated samples.

XIV $${\mathrm{Cl}}^{-}+2{\mathrm{OH}}^{-}\to \mathrm{Cl}O^{-}+H_{2}O+2e^{-}$$

XV $$\mathrm{Cl}O^{-}+H^{+}\to \mathrm{HClO}$$

XVI $${\mathrm{Cl}}^{-}+2H_{2}O\to \mathrm{Cl}O_{2}+4H^{+}+5e^{-}$$

XVII $$2{\mathrm{Cl}}^{-}+H_{2}O\to {\mathrm{Cl}}_{2}O+2H^{+}+4e^{-}$$

XVIII $$2H_{2}O+2e^{-}\to H_{2}+2{\mathrm{OH}}^{-}$$

XIX $${\mathrm{Na}}^{+}+{\mathrm{OH}}^{-}\to \mathrm{NaOH}$$

Therefore, since Na^+^ and Cl^–^ are transformed into subproducts with lower electronic availability, it is expected that conductivity will be reduced due to this. In addition, higher salt levels contribute to the formation of emulsions.^47^ Thus, after the electrochemical reactor, it can be seen that the emulsions were partially broken (Figure 2). So, after the strainer, the aqueous outlet (point C) showed lower turbidity (Figure 2), to a certain extent, due to emulsion destabilization and an increase in the solubilization of the compounds in the aqueous current. However, it was also observed that the presence of solids at the bottom of the point B jar after it stood still for some time (Figure 2). Thereby, it is probable that solids coagulated with one another and then separated by gravitational forces in the strainer. Thus, as turbidity is highly related to suspended solids, since they were removed, this also contributed to the decrease in turbidity.

Furthermore, O&G content at step B presented a similar value to the initial sample, for 2 and 4 RT, indicating slight oxidation at this point and under these conditions. However, for these times, at points C and D, it decreased substantially, representing a removal of at least 80%. Yet, when 8 min of RT was applied, the TOG removal was high already at point B (60%), followed by further removal as observed for the other samples. It is important to highlight that all samples at the final disposal outlet (point D) achieved low enough TOG content (1–22 mg·L^–1^) to be discharged in accordance with several countries’ laws.

Taking these facts into consideration, some hypotheses can be raised: (i) oxidizing radicals are formed in the reactor and continue to react in the strainer, which has a longer RT, four times higher, to be more precise. Thus, when the RT at the reactor was increased, the radicals had enough time for the reaction to be noticeable into the reactor rather than on the strainer; (ii) an EC/electroflotation process is also observed with some solids decanting at the bottom of the strainer, and, at the same time, a supernatant oily phase was formed at point B, which was separated by flotation by the gases previously formed on the reactor being the bubble formation being favored by the pressure drop within the strainer; and (iii) a partial rupture of the emulsion occurs, not only because of the salinity reduction (as already discussed) but also due to the reduction of the superficial charge of the droplets, causing the coalescence of the oil droplets and the subsequent separation of the phases, which normally is associated with the presence of metal salts.^48^

After the TOG analysis, the residual O&G from S0 and S2-C were resolubilized and analyzed by GC/MS (Figure 3). It becomes clear that the initial sample (S0) presented several different compounds, which were mostly low-mass compounds (short retention time), although some medium-high mass (long retention time due to their more intense interactions with the stationary phase, moving more slowly through the column) compounds were also detected. On the other hand, in the S2-C sample, almost no low-mass compounds were identified, while the medium-high-mass compounds had low reductions or remained practically the same.

**Figure 3:**
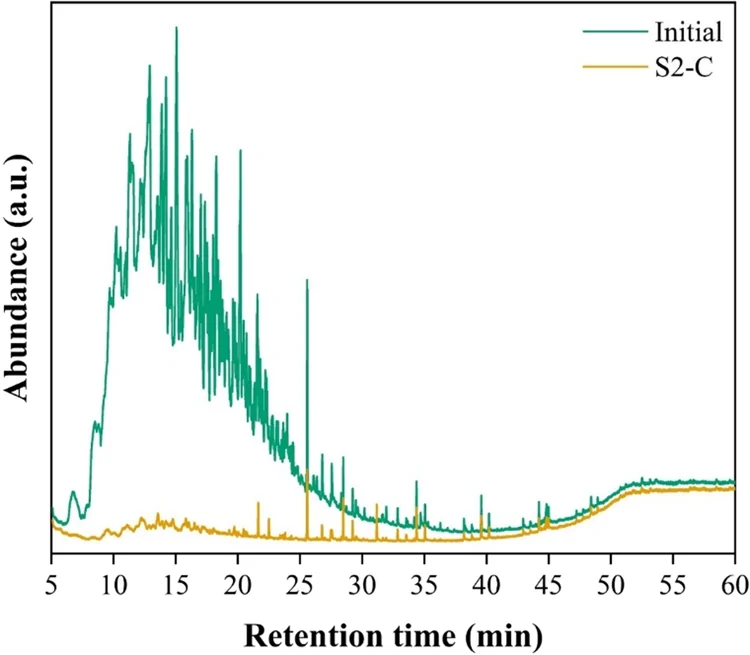
CG-MS spectrum from the untreated (initial) and treated after the strainer (S2-C, strainer outlet) samples redissolved after the TOG extraction. Experimental conditions: CD = 25 A·m^–2^; RT = 4 min, pH = natural, temperature = room temperature.

This behavior could be related to the following: (i) the electrochemical treatment was more efficient for molecules with higher solubility, which can be correlated to the characteristics of the pollutants (hydrophilicity, molecular orbital energies, electronic charge, functional molecular groups, among others), the pollutant-electrodes’ surface tension compatibility, the hydrophilic behavior of the oxidative radicals, and the facilitated mass transfer mechanisms;^49^ (ii) NA, which are more commonly detected from 2 to 24 carbons,^50^ i.e., low molecular mass dissolved oils, are probably the major category present in this OPW sample, since no TPH (dispersed oil) was detected on the initial specimen. Therefore, decarboxylation is a probable oxidation mechanism, enabling easy gravitational separation due to polarity differences;^43^ (iii) subproducts that are not quantified in the TOG analysis might be formed. The EO can generate subproducts less susceptible to *n*-hexane extraction or with reduced boiling point (volatilizing at 85 °C); and (iv) EC can contribute to the flocculation of the dissolved oils,^51^ trapping these pollutants on the produced sludge, which is segregated by flotation/sedimentation on the strainer.

Combining the visual observations with the physicochemical, chromatographic, and toxicity analyses, the electrochemical treatment of offshore OPW was shown to involve a hybrid mechanism combining EO, EC, adsorption/coagulation, and flotation. As pointed out by Danaee et al.,^52^ it is really hard to fully disentangle these mechanisms, as several times they occur concomitantly. Therefore, the proposed mechanistic insights described here should be interpreted as an experimentally supported mechanism rather than as a fully decoupled demonstration of each individual pathway.

EC contributed to charge destabilization, the aggregation of dissolved and dispersed organic compounds, and floc formation. The presence of adsorption and coagulation associated with it is supported by the formation of visible solids/flocs, a shift in ζ-potential toward values near the isoelectric region, an increase in Al and Zn concentrations after treatment, and a sharp reduction in turbidity and TOG after the strainer. These phenomena have already been described in the literature, which are related to the formation of dissolved metal ion complexes and metal hydroxides in water, causing suspended particles to aggregate (via the action of surface charge) and precipitate/adsorb dissolved pollutants.^53^

At the same time, gas-assisted flotation and phase separation are supported by gas evolution inside the reactor (mainly associated with O_2_, Cl_2_, and H_2_ formation), the pressure drop in the strainer, the visual formation of a second liquid phase at the reactor outlet (Figure 2), and the decrease in the TOG between the reactor outlet and the strainer outlet. Therefore, it can be concluded that these physicochemical processes also play an important role in the reaction mechanism.

On the other hand, EO promoted the partial transformation of dissolved organic compounds, including NA, likely via decarboxylation, yielding products that were less polar and less soluble in the aqueous phase. The oxidative transformation is evidenced by the reduction in the TOC rate, a modified GC/MS profile after treatment, and the disappearance or substantial reduction of low-retention-time organic compounds in the treated sample.

To understand and quantify the contribution of each of these processes in the treatment, it would be necessary to evaluate them separately, as already reported in previous publications.^52–54^ However, in the case of this study, to fully distinguish adsorption/coagulation, flotation, phase separation, and true oxidative degradation pathway, this would require not only other pilot plants’ configurations as well as a significantly larger volume of OPW sample but also, taking into consideration the complexity of the composition of the real OPW, carbon mass balance, characterization of the sludge, aqueous, and floated organic phases, identification of oxidation intermediates, and oxidant-quenching experiments would be infeasible and would also lead to inaccurate results. Therefore, although the mechanism proposed here does not provide evidence for each step individually, it should still be regarded as a plausible interpretation supported by multiple indirect and complementary pieces of evidence.

It is also important to highlight here that although this study focused on RT and scale-up performance, CD and pH are recognized as key variables in the electrochemical OPW treatment. Increasing CD can enhance EC, gas-assisted flotation, and anodic and/or indirect oxidation, thereby influencing the TOG removal and the degradation of dissolved organic matter.^19,55^ However, excessive CD increases energy consumption, heat generation, electrode abrasion, gas evolution, and the formation of potentially toxic inorganic chlorine-derived or organochlorinated byproducts.^19,55^ Likewise, pH influences the metal hydroxide speciation, floc stability, surface charge, NA solubility, and active chlorine speciation.^5^ However, operation at a natural near-neutral pH was selected as a practical condition for offshore application, avoiding additional chemical consumption and operational complexity. Nevertheless, future studies to systematically evaluate the CD and pH influences on the process performance are encouraged.

Furthermore, S0, S2-C, and the chemical treatment samples were meticulously analyzed. The results are listed in Table 4. It is noticeable that both the electrochemical and the chemical routes were able to reduce the parameter total organic carbon (TOC), chloride, titanium, radium-226, radium-228, and free chlorine. However, the advanced treatment proved to be more effective, reaching limits below the quantification limit for most of these parameters.

**Table 4:** Composition and Physicochemical Characterization of the Initial Real OPW (S0), after the Advanced Treatment (S2-C, Strainer Outlet), and after Chemical Route Treatment^a^

	sample			
compound	S0	S2-C	chemical treatment	QL^b^
ammoniacal nitrogen (mg·L^–1^)	0.109	<0.05	0.101	0.050
total organic carbon (mg·L^–1^)	561	42	180	1.0
chloride (g·L^–1^)	30	5	25	0.50
total phenols (mg·L^–1^)	<0.100	<0.100	<0.100	0.100
mercury (mg·L^–1^)	<0.20	<0.20	<0.20	0.20
arsenic (μg·L^–1^)	<8.0	<8.0	<8.0	8.0
cadmium (μg·L^–1^)	<1.0	<1.0	<1.0	1.0
lead (μg·L^–1^)	<10.0	<10.0	<10.0	10.0
copper (μg·L^–1^)	<5.0	<5.0	<5.0	5.0
chrome (μg·L^–1^)	<5.0	<5.0	<5.0	5.0
iron (μg·L^–1^)	448	417	437	7.0
manganese (μg·L^–1^)	28	27.1	35	5.0
nickel (μg·L^–1^)	<6.0	<6.0	<6.0	6,0
zinc (μg·L^–1^)	21	78	27	5.0
aluminum (μg·L^–1^)	218	1304	206	10.0
barium (mg·L^–1^)	11	2	11	1.0
sodium (mg·L^–1^)	954	661	954	200.0
titanium (μg·L^–1^)	171	<5.0	<5.0	5.0
vanadium (μg·L^–1^)	<5.0	<5.0	<5.0	5.0
naphthalene (μg·L^–1^)	<0.012	<0.012	<0.012	0.012
acenaphthylene (μg·L^–1^)	<0.012	<0.012	<0.012	0.012
anthracene (μg·L^–1^)	<0.012	<0.012	<0.012	0.012
benzo(a)anthracene (μg·L^–1^)	<0.012	<0.012	<0.012	0.012
benzo(a)pyrene (μg·L^–1^)	<0.012	<0.012	<0.012	0.012
benzo(b)fluoranthene (μg·L^–1^)	<0.012	<0.012	<0.012	0.012
benzo(g,*h*,i)perylene (μg·L^–1^)	<0.012	<0.012	<0.012	0.012
benzo(k)fluoranthene (μg·L^–1^)	<0.012	<0.012	<0.012	0.012
chrysene (μg·L^–1^)	<0.012	<0.012	<0.012	0.012
dibenzo(a,h)anthracene (μg·L^–1^)	<0.012	<0.012	<0.012	0.012
phenanthrene (μg·L^–1^)	<0.012	<0.012	<0.012	0.012
fluoranthene (μg·L^–1^)	<0.012	<0.012	<0.012	0.012
fluorene (μg·L^–1^)	<0.012	<0.012	<0.012	0.012
indene(1,2,3,*c*-d)pyrene (μg·L^–1^)	<0.012	<0.012	<0.012	0.012
1-methylnaphthalene (μg·L^–1^)	<0.012	<0.012	<0.012	0.012
2- methylnaphthalene (μg·L^–1^)	<0.012	<0.012	<0.012	0.012
3-methylchloranthrene (μg·L^–1^)	<0.015	<0.015	<0.015	0.015
pyrene (μg·L^–1^)	<0.012	<0.012	<0.012	0.012
*n*-octane (C8) (μg·L^–1^)	<2.5	<2.5	<2.5	2.5
*n*-nonane (C9) (μg·L^–1^)	<2.5	<2.5	<2.5	2.5
*n*-decane (C10) (μg·L^–1^)	<2.5	<2.5	<2.5	2.5
*n*-undecane (C11) (μg·L^–1^)	<2.5	<2.5	<2.5	2.5
*n*-dodecane (C12) (μg·L^–1^)	<2.5	<2.5	<2.5	2.5
*n*-tridecane (C13) (μg·L^–1^)	<2.5	<2.5	<2.5	2.5
*n*-tetradecane (C14) (μg·L^–1^)	<2.5	<2.5	<2.5	2.5
*n*-hexadecane (C16) (μg·L^–1^)	<2.5	<2.5	<2.5	2.5
*n*-heptadecane (C17) (μg·L^–1^)	<2.5	<2.5	<2.5	2.5
*n*-octadecanoe(C18) (μg·L^–1^)	<2.5	<2.5	<2.5	2.5
*n*-nonadecane (C19) (μg·L^–1^)	<2.5	<2.5	<2.5	2.5
*n*-eicosane (C20) (μg·L^–1^)	<2.5	<2.5	<2.5	2.5
*n*-heneicosane (C21) (μg·L^–1^)	<2.5	<2.5	<2.5	2.5
*n*-docosane (C22) (μg·L^–1^)	<2.5	<2.5	<2.5	2.5
*n*-tricosane (C23) (μg·L^–1^)	<2.5	<2.5	<2.5	2.5
*n*-tetracosane (C24) (μg·L^–1^)	<2.5	<2.5	<2.5	2.5
*n*-pentacosane (C25) (μg·L^–1^)	<2.5	<2.5	<2.5	2.5
*n*-hexacosane (C26) (μg·L^–1^)	<2.5	<2.5	<2.5	2.5
*n*-heptacosane (C27) (μg·L^–1^)	<2.5	<2.5	<2.5	2.5
*n*-octacosane (C28) (μg·L^–1^)	<2.5	<2.5	<2.5	2.5
*n*-nonacosane (C29) (μg·L^–1^)	<2.5	<2.5	<2.5	2.5
*n*-triacontane (C30) (μg·L^–1^)	<2.5	<2.5	<2.5	2.5
*n*-hentriacontane (C31) (μg·L^–1^)	<2.5	<2.5	<2.5	2.5
*n*-dotriacontane (C32) (μg·L^–1^)	<2.5	<2.5	<2.5	2.5
*n*-tritriacontane (C33) (μg·L^–1^)	<2.5	<2.5	<2.5	2.5
*n*-tetratriacontane (C34) (μg·L^–1^)	<2.5	<2.5	<2.5	2.5
*n*-pentatriacontane (C35) (μg·L^–1^)	<2.5	<2.5	<2.5	2.5
*n*-hexatriacontane (C36) (μg·L^–1^)	<2.5	<2.5	<2.5	2.5
*n*-heptatriacontane (C37) (μg·L^–1^)	<2.5	<2.5	<2.5	2.5
*n*-octatriacontane (C38) (μg·L^–1^)	<2.5	<2.5	<2.5	2.5
*n*-nonatriacontane (C39) (μg·L^–1^)	<2.5	<2.5	<2.5	2.5
*n*-tetracontane (C40) (μg·L^–1^)	<2.5	<2.5	<2.5	2.5
fitane (μg·L^–1^)	<2.5	<2.5	<2.5	2.5
pristane (μg·L^–1^)	<2.5	<2.5	<2.5	2.5
*n-*pentadecane (C15) (μg·L^–1^)	<2.5	<2.5	<2.5	2.5
total PAHs^c^ (μg·L^–1^)	<0.25	<0.25	<0.25	0.25
TPH^d^ - diesel range (C11–C28) (μg·L^–1^)	<50	<50	<50	50.0
TPH^d^ - oil range (C20–C36) (μg·L^–1^)	<50	<50	<50	50.0
TPH^d^ - kerosene range (C11–C14) (μg·L^–1^)	<10	<10	<10	10
TPH^d^ - gasoline range (C5- C10) (μg·L^–1^)	<25	<25	<25	25.0
total TPH^d^ (C5–C40) (μg·L^–1^)	<125	<125	<125	125
benzene (μg·L^–1^)	<0.5	<0.5	31.09	0.50
toluene (μg·L^–1^)	<0.50	<0.50	36.3	0.50
ethylbenzene (μg·L^–1^)	<0.50	<0.50	4.2	0.50
xylenes (μg·L^–1^)	<1.50	<1.50	6.17	1.50
radium-226 (Bq·L^–1^)	2	<0.44	1.5	0.44
radium-228 (Bq·L^–1^)	0.36	<0.32	<0.32	0.32
free chlorine (mg·L^–1^)	0.44	0.06	0.33	0.05

a Experimental conditions: CD = 25 A·m^–2^; RT = 4 min, pH = natural, temperature = room temperature.

b Quantification limit.

c Polycyclic aromatic hydrocarbons (PAHs).

d Total petroleum hydrocarbons.

In addition, the electrochemical process was also able to considerably reduce the concentrations of ammoniacal nitrogen, barium, and sodium. These high removal rates are linked to the oxidation (anode) and reduction (cathode) reactions that occur on the surface of the electrodes, managing to remove a wide range of compounds concomitantly and quickly.^56^

The other parameters remained nearly unchanged and were mostly below the detection limit. Nevertheless, it is worth mentioning that the sample that underwent the conventional treatment showed higher values of benzene, toluene, ethylbenzene, and xylene (BTEX) than the initial ones. However, the arising of these compounds is inconsistent with the procedure applied, that is, lowering the pH with acetic acid (facilitating phase separation due to the lower solubility of the oils in this condition),^56^ followed by the addition of flocculant and flotation. In other words, in this phase, chemical reactions that would lead to the breakdown of larger molecules or the conversion of smaller molecules into byproducts are unlikely. Thus, it is concluded that cross-contamination occurred during the test, leading to the appearance of these compounds in μg·L^–1^ concentration order.

In contrast, the concentrations of Al and Zn are the only ones that increase after electrochemical treatment, with both materials having already been reported as electrocoagulants in the literature.^3,57^ This suggests that the reaction system, in fact, combines EO and EC, achieving efficient and rapid removal by combining these processes and then separating the coagulates and foam formed in the gravitational separator.

Finally, the high reduction in the levels of salinity, sodium, chloride, and free chlorine is important, since these results reinforce the conclusions discussed before, related to the electrochemical reactions affecting these properties. Furthermore, it is important to highlight that all measured parameters at the end of the proposed treatment are in accordance with Brazil’s wastewater discharge standards.^35^

Once no free oil was observed in the initial sample, the effect of the ζ potential behavior was mainly determined by the soluble components in the effluent, such as salt, electrolytes, dissolved oil compounds, surfactants, and other chemical additives.^58^ ζ-potential showed a negative initial value of – 9.17 mV (Figure 4). However, after the electrochemical reactor, its value turned out to be positive (3.42 mV). Therefore, it can be concluded that at some point, the supplied electric charges caused the system to achieve its isoelectric point. This phenomenon promotes the reduction of the repulsive electrostatic forces, leading to a quick coalescence of the oil droplets from the oil/water emulsion.^59^ Additionally, Fan et al.^59^ also found that viscoelastic and electrical properties of oil/water emulsions could be affected by the electro-oxidation of organic surfactants, destabilizing the charges in the medium, causing the rupture of the oil droplets’ interfacial films, and favoring the oil droplets’ aggregation as a consequence.

**Figure 4:**
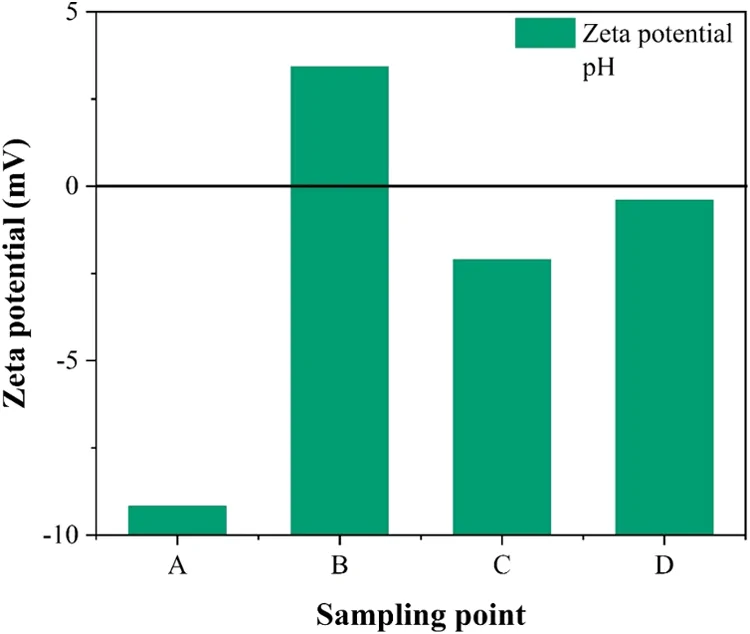
ζ-potential for initial (S0, raw OPW) and treated in Unit 1 (S2–B, electrochemical reactor outlet; S2–C, strainer outlet; and S2-D, filter outlet) OPW at different sampling points. Experimental conditions: CD = 25 A·m^–2^; RT = 4 min, pH = natural, temperature = room temperature.

On the other hand, after the strainer and the filter, the ζ potential becomes negative again (−2.10 and −0.39 mV, respectively); however, with values very close to zero, thus demonstrating the emulsion’s instability, which is related to the surfactants’ oxidation along with the salinity reduction.^58,59^

As shown in Figure 5A, acute toxicity was slightly reduced by 4 min of RT at points C and D. However, the same did not occur for 2 and 8 min residence times. Under these conditions, EC50 decreased considerably, i.e., presents higher toxicity, which is correlated to the subproducts formed. Several studies demonstrated that the generation of more harmful intermediates may occur during oxidation reactions, needing an optimal stage to be discovered for the reaction to be stopped, since, in general, complete mineralization cannot be achieved, or it is not economically viable.^60,61^

**Figure 5:**
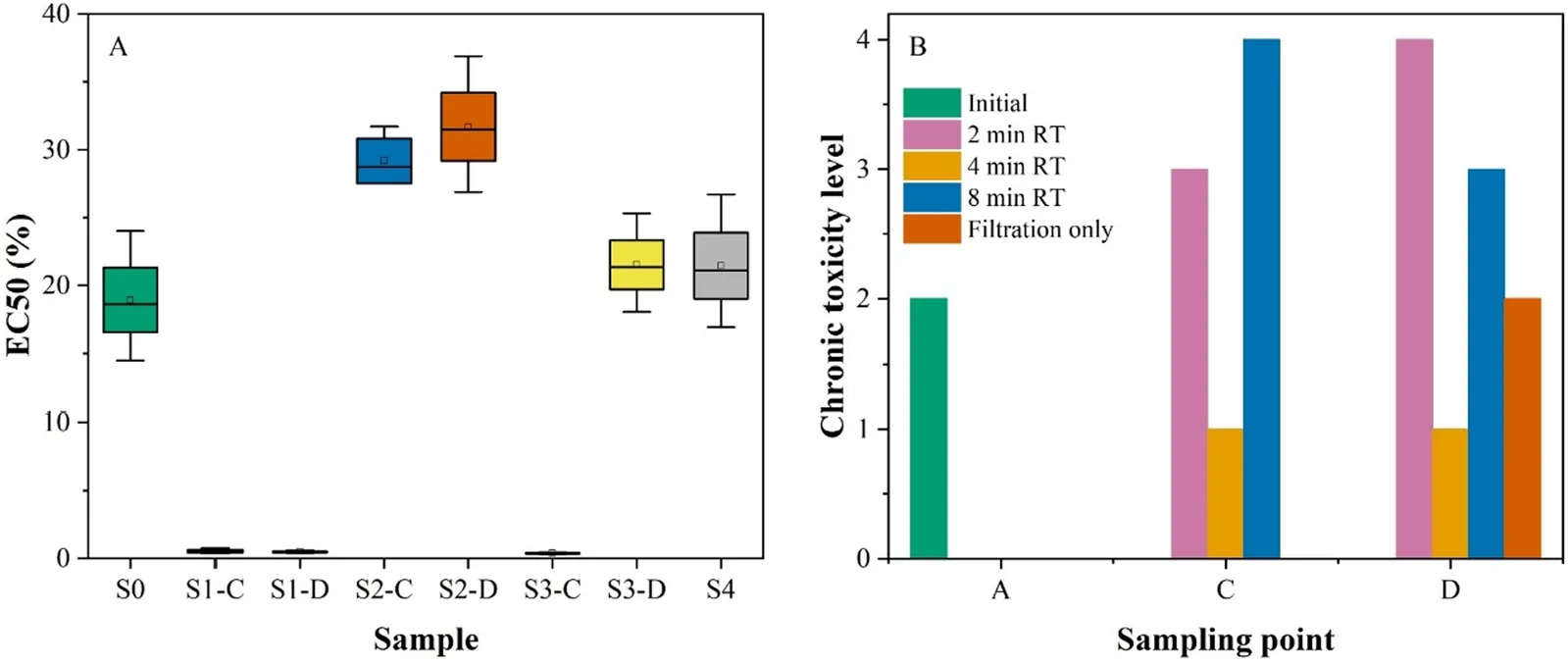
Acute toxicities (A) and chronic toxicities (B) from tests conducted in Unit 1, where S0 is the initial OPW sample, S1, S2, and S3 represent 2, 4, and 8 min of RT, respectively, S4 is the filtration only sample, and A is the feeding point, and C and D correspond to the sampling points on the strainer and filter outlets, respectively.

Similarly, chronic toxicity (Figure 5B) demonstrated similar behavior with a decrease in the toxicity to level 1 when 4 min RT was used and an increase to levels 3 or 4 when other times were applied. This reinforces the considerations discussed in the previous paragraph.

These results are extremely relevant, since one of the major concerns in operating electrochemical processes under hypersaline environments is the generation of inorganic chloride species (HClO, ClO^–^, ClO_2_^–^, ClO_3_^–^, and ClO_4_^–^) and organochlorines.^55^ Thus, these findings provide evidence that, even though these compounds were probably formed, they were still in concentrations low enough not to prejudice the acute and chronic toxicity. This behavior is connected to the reduced RTs applied, which do not give enough time for these species to accumulate. In this sense, 4 min of RT has been demonstrated to be the most promising condition for the next investigations, thus being the time selected to be applied in the larger Unit 2 pilot-scale unit.

### 3.2 Scale-Up Efficiency Comparison

As can be observed in Figure 6, all analyses showed similar or even better results than those obtained for Unit 1, demonstrating a successful scale-up. In fact, the reductions of acute toxicity and TOG content excelled. In particular, this last one, which demonstrated a high decrease already at point B, achieved 15 mg·L^–1^ with 4 min of RT and 25 A·m^–2^, as applied previously in Unit 1. This difference in effectiveness is associated with the optimized internal design of the bigger reactor.^16,17^ However, it is important to highlight that chronic toxicity increased by one level in Unit 2, presenting similar responses to the untreated sample. Therefore, operational parameters need to be further optimized to improve this factor to values close to those reported for the smaller unit.

**Figure 6:**
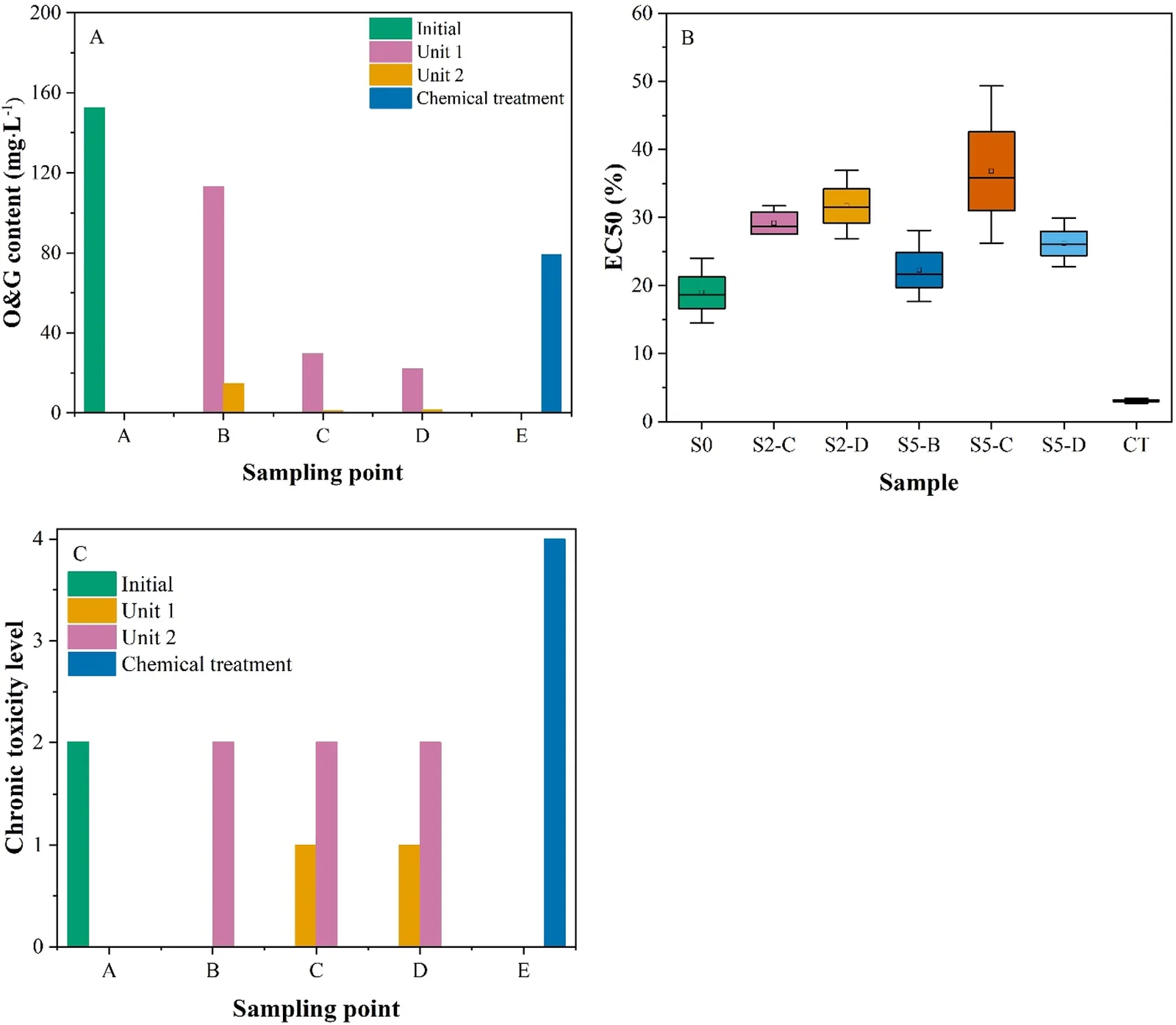
O&G content (A), acute toxicities (B), and chronic toxicities (C) of the collected samples, where S0 is the initial sample, S2 and S5 represent the experiments performed on Units 1 and 2, respectively, and A is the feeding point, and, B, C, and D correspond to the sampling points on the electrochemical reactor, strainer, and filter outlets, respectively. Experimental conditions: CD = 25 A·m^–2^; RT = 4 min, pH = natural, temperature = room temperature.

Comparing these results with those of conventional treatment, it is possible to observe that conductivity and salinity were practically unaffected (Figure 7). However, the pH was slightly reduced because of the methodology used, which lowers the pH to 5 before flotation. Turbidity also reduced but considerably less than with the electrochemical process, which was visually more turbid.

**Figure 7:**
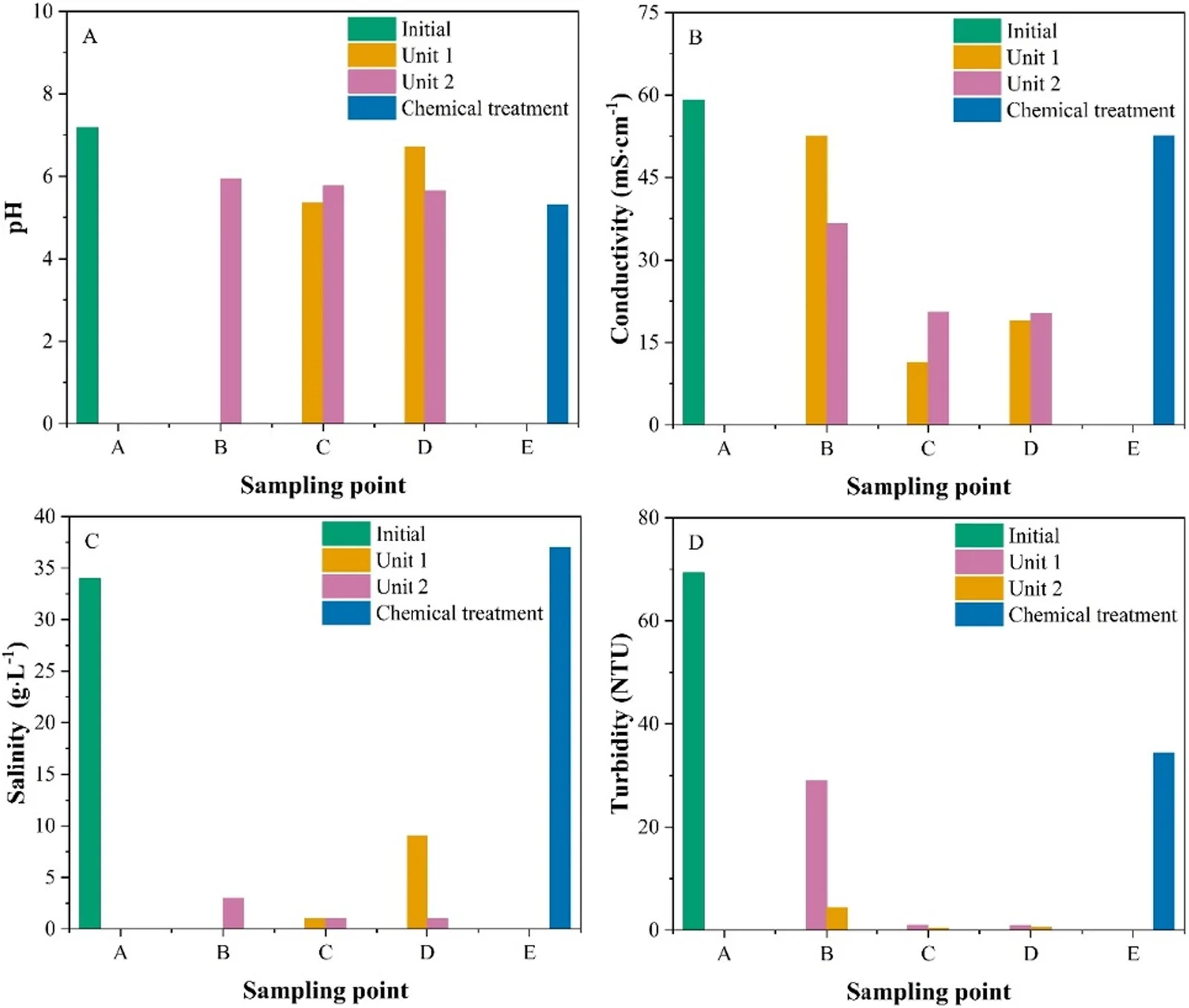
pH (A), conductivity (B), salinity (C), *and* turbidity (D*)* of the collected samples, where S0 is the initial sample, S2 and S5 represent the experiments performed on Units 1 and 2, respectively, and A is the feeding point, and, B, C, and D correspond to the sampling points on the electrochemical reactor, strainer, and filter outlets, respectively. Experimental conditions: CD = 25 A·m^–2^; RT = 4 min, pH = natural, temperature = room temperature.

However, the most interesting responses were related to the most important parameters for effluent disposal: TOG, acute toxicity, and chronic toxicity. In all of these cases, treatment using an advanced process proved to be much more effective. Not only did it reduce toxicity but also it achieved TOG values suitable for discharge. It is relevant to emphasize at this point that the chemical treatment procedure carried out in this study attempts to simulate real conditions. Nonetheless, the equipment used in the laboratory was very simple; thus, unfortunately, it did not represent the real conditions found in industrial systems such as considerably lower temperatures.

Moreover, COD, solid analysis, and superficial tension were also performed to investigate the mechanism of the reaction further (Figure 8). First of all, like the abovementioned, Unit 2 demonstrated a similar behavior to the results observed in Unit 1. The major difference is noticed at point B, which can be attributed, once again, to a more efficient design of the Unit 2 reactor, causing the reactions to occur more rapidly.^16,17^

**Figure 8:**
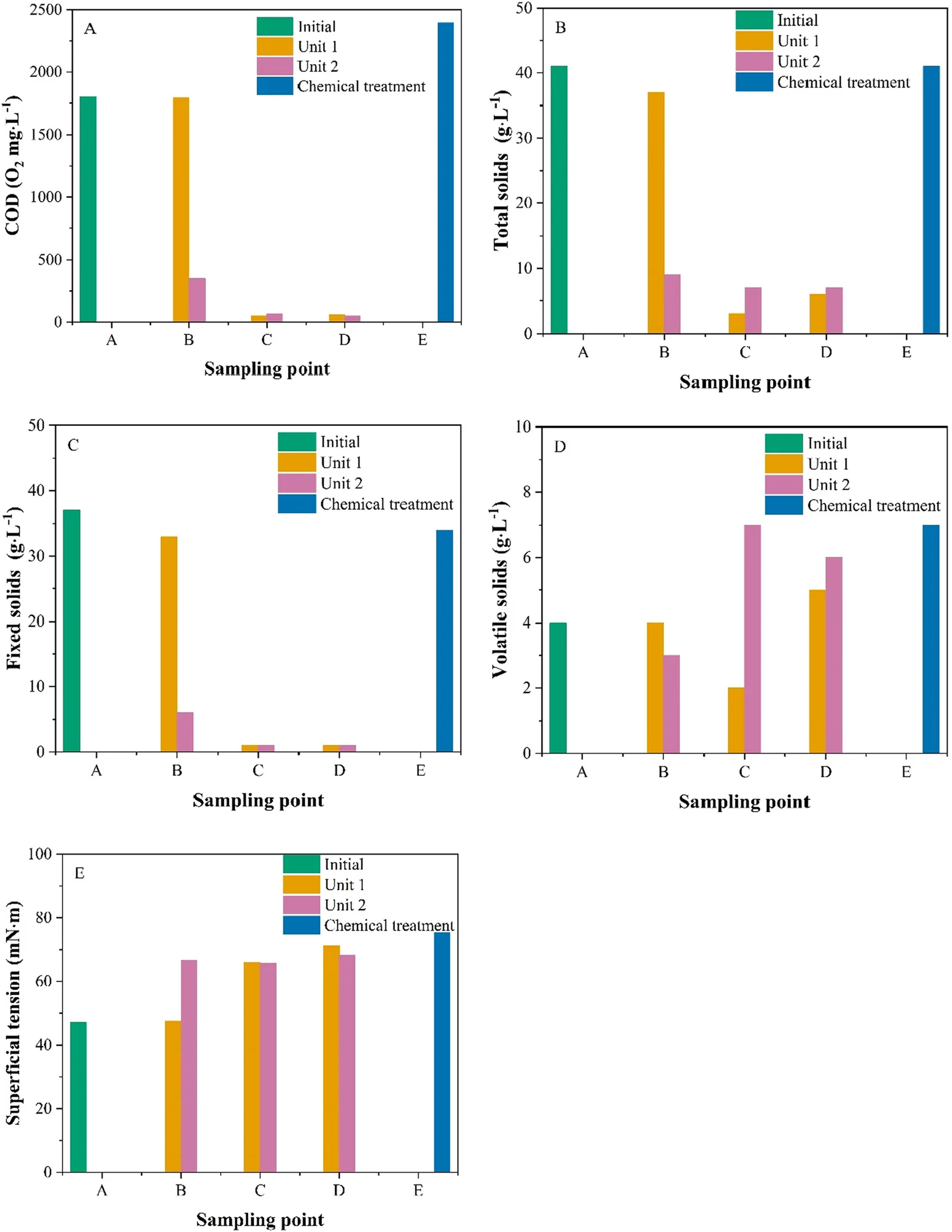
COD (A), total solids (B), fixed solids (C), volatile solids (D), and superficial tension (E) results from Unit 2, where A is the feeding point, and B, C, and D correspond to the sampling points on the electrochemical reactor, strainer, and filter outlets, respectively. Experimental conditions: CD = 25 A·m^–2^; RT = 4 min, pH = natural, temperature = room temperature.

Furthermore, it can be noticed that COD, total solids, and fixed decreased in similar proportions throughout the processes (Figure 8A–C). This is correlated with the already reported salinity reduction (Figure 7C), since, as discussed previously, high salinities favor oil/water emulsions to be formed.^58,59^ Thus, reducing this factor contributes to emulsion segregation. Besides this fact alone that would reduce COD rates (especially at points C and D where higher oxidation levels were achieved), Cl^–^ anions, which contribute to COD augmentation, were partially or totally oxidized by electrochemical oxidation (HClO, Cl_2_O, or ClO_2_) or transformed into Cl_2_ gas.^44,46^ Therefore, it also leads to a decrease in COD. Moreover, electro-oxidation of inorganic compounds, such as heavy metals, nitrates, and sulfates, contributes to the COD reduction.^56^

However, the sample treated by the chemical route did not show significant changes. An increase in COD due to the addition of the flocculant was observed due to the increase in the soluble organic load, although it did not contribute to the increase in TOG.

At the same time, two main factors influenced the total and fixed solid removal. After the reactor, not only smaller and more volatile compounds were formed by EO (not being quantified as total and fixed solids), but also electrocoagulated solids were segregated in the strainer. In addition (Figure 8D), volatile solids presented a 75% increase in the strainer outlet of unit 2, reinforcing that the radicals formed in the electrochemical system continue to react within the strainer, providing additional oxidation of the molecules.

The comparison between the 6 and 15 L pilot units showed that the electrochemical process could be operated in a larger pilot configuration while maintaining or improving the treatment performance under the tested conditions. However, this result should not be interpreted as evidence of a direct scale-up based on solely the reactor volume. The two units differed not only in volume but also in electrode area, applied voltage, electrical charge, and energy input. Therefore, the improved performance observed in unit 2 may be attributable to a combination of all of these factors, including a higher available electrode surface area, different electrode area-to-volume and electrode area-to-flow ratios, greater electrochemical charge supplied to the system, and an improved reactor design. Thus, the results support the feasibility of larger unit operations and provide relevant information for scale-up-related assessment, but they do not establish a complete scale-up law. Further investigations are still needed not only to define the optimal geometric, hydrodynamic, electrical, and energetic configuration of the reactor but also to refine its operating conditions, in terms of energy consumption, heat generation, gas evolution, byproduct formation, and required footprint, before full-scale offshore implementation.

Additionally, superficial tension changed significantly after the reactor, raising from 47 to 66 mN·m^–1^ (Figure 8E). This fact evidence the degradation from surfactants, which cause intermolecular forces between the water molecules and/or other pollutants interfacial forces to decrease by electronic stabilization or by steric effect.^62^ Aside from that, high-salinity levels contribute to superficial tension to be reduced. Salt solutions possess ion-specific effects provoking attraction or repulsion at the oil–water interface. Na^+^ may be attracted toward a negatively charged interface, thus enhancing its solubility in the oil phase. Therefore, high polarizability and low hydration would predominate over the Cl^–^ repulsion.^62^ Moreover, at the end of the treatment (point D), superficial tension reached 68–71 mN·m^–1^, which is very close to the natural water value (73 mN·m^–1^).^63^ On the other hand, values close to those of seawater were found for the chemically treated sample (75 mN·m^–1^),^64^ again showing that this sample is much closer in characteristics to the real OPW than the electrochemically treated one.

Thus, it has been shown that commercial electrochemical pilot-scale systems emerge as a promising solution for the treatment of real OPW. Therefore, further investigations *in loco* at oilfield platforms should be carried out not only to develop the TRL of the process but also to give a deeper understanding of operational and long-term running challenges.

Since the results obtained both on bench and pilot scales were promising, this encourages applications on even greater scales, up to the industrial scale. However, some issues that are not usually taken into consideration in smaller units become relevant for a large-flow treatment. In particular, electrochemical supplies, energy consumption, heat generation, gas volume outputs, and financial costs are points of attention.^19,55^ Furthermore, for offshore operations, footprint and weight are also essential characteristics for new technology implementation.^2^ Therefore, these parameters were calculated for the tested electrochemical plants (Table 5).

**Table 5:** Technoeconomic Calculated Parameters for the Electrochemical Pilot Plants in Two Different Scales (Units 1 and 2)

plant scale	bench (unit 1)	pilot (unit 2)
reactor’s volume (L)	6	15
flow (m^3^·h^–1^)	0.09	0.22
RT (min)	4	4
CD (A·m^–2^)	25	25
intensity	1.5	13.0
electrical charge (Ah·m^–3^)	17	59
geometrical area of the cells/volume of reactor (m^–1^)	10	35
geometrical area of the cells/flow rate (h·m^–1^)	0.7	2.3
*W* (W)	7	260
*E*_C_ (kWh kg of TOG removed^–1^)	0.5	7.8
EEC (USD·m^–3^)	0.01	0.13
H_2_ generation (L·h^–1^)	0.6	5.4
O_2_ generation (L·h^–1^)	0.3	2.7
Cl_2_ generation (L·h^–1^)	0.6	5.4
*q* (W)	5	166
*m*_W_ (g·h^–1^)	8	265
*F*_A_ (m·h^–1^)	6	10
*F*_V_ (h^–1^)	15	15

First of all, the calculated electrical charge as well as the geometrical area of the cells/volume of reactor and geometrical area of the cells/flow rate ratios were approximately triple for Unit 2. This information helps to explain the higher TOG removal performance observed in the bigger unit, since not only a higher surficial area and active sites consequently were available but also more electrical energy was provided per treated m^3^, which could lead to an increase on the ROS generation rate, hence promoting a faster TOG removal.

Moreover, comparing the results obtained, it can be observed that the electrical power (W) required and the specific energy consumption (*E*_C_) for the major unit are 37× and 16× times, respectively, higher than for the smaller unit, even though the flow treated is only 2.4× higher in the greater unit. This is probably related to energy losses on the longer cells, less uniform superficial electric dispersion, and greater distance between the cells.^19,55^

Additionally, typical produced water costs vary from 19 to 188 USD·m^–3^, with specific costs for basic separation, varying from 0.44 to 0.75·USD m^–3^ without considering storage costs.^65,66^ Thus, when only EEC is considered, the obtained values are within a reasonable range compared with other separation techniques. It is important to highlight that operation costs will be higher, considering that deposition of oxidation products, adsorption of organic matter, formation of mineral scales, and solids removal and disposal are predicted.^16,17^ Therefore, regular cleaning and maintenance, periodic electrode replacement, and solids management will be required for a successful operation. These facts evidence the importance of investigating the OPW treatment by electrochemical pilot plants, since unexplored points still have to be evaluated before full-scale operations.

That being said, energetic power supplementation remains the biggest challenge to be overcome for this technological implementation on a large scale since a considerable amount of energy at a harsh intensity is demanded. Therefore, operational, financial, and security aspects still need further in-depth evaluations. In particular, taking into consideration that real OPW flows can reach much higher proportions.^67^

Another point of attention is that, since voltage did not rise linearly with the treated flow, its considerable increase caused a heat generation close to 33× times higher in the pilot scale when compared to the bench scale. This suggests that heat generation for larger units might increase considerably, making it hard to extrapolate data from the collected data from the pilot scale. Also in this sense, an industrial offshore plant operates at high temperatures;^67^ thus, not only proper materials for the reactor’s carcass and connection constructions need to be evaluated but also the volume of the water vapor formed/exhausted needs to be further analyzed during longer operation times.

Moreover, considerable volumes of H_2_, O_2_, and Cl_2_ (0.3 to 5.4 L·h^–1^) are likely to be produced. Therefore, for industrial-scale applications, a proper gas disposal system (gas scrubbers, filters, exhaust fans, burners, among others) associated with it would be required. Also, it is important to take into consideration that H_2_ has explosive and flammable potential depending on its concentration. Moreover, Cl_2_ in high concentrations has a toxic and corrosive behavior. In this sense, its exposure controls and adequate retention (normally by gas scrubbers with NaOH solution) are essential to ensure the system’s operation safety. Thus, security and classified area adjustment may be needed for this equipment operation on offshore facilities, requiring appropriate ventilation, gas exhaustion, and safeguards (alarms, materials, interlocks, among others). On the other hand, H_2_ is also a high-performance fuel. Therefore, if it can be segregated from the other gases, it can be stored and applied for energy production, reducing operational costs for energy supply.

Still, in the sense of cost reductions, EO can produce HClO from saline water (eq 15), and EC generates an electrocoagulant inherently to the process. Thereby, subproducts from the electrochemical treatment could be taken advantage of, replacing chemical additives such as disinfectants and surfactants/flocculants/coagulants.

In addition, the footprint parameter for both units is equivalent to that of other equipment already in operation on offshore units, which in some cases can be even more relevant than financial considerations for real implementation. Gravity sedimentation and air flotation operations, for example, present F_A_, respectively, of 4 m·h^–1^ and 1 h^–1^ and F_V_ of 2–16 m·h^–1^ and 4–15 h^–1^, in the same order.^2^ The data from the studied electrochemical system indicate FA and FV values of 10 m·h^–1^ and 15 h^–1^, respectively, for the Unit 2. This implies that electrochemical reactor capacities could be scaled up to operate in areas and volumes equivalent to those of air flotation columns. In fact, the proposed system could work in electrochemical reactor modules. Therefore, considering the RT employed, five reactors would be required to treat 1 m^3^·h^–1^ of OPW. Furthermore, these results for the pilot plant were even better than those for the bench scale, which is relevant for offshore applications, since reduced horizontal area is essential for new operations implementation.

Furthermore, it is relevant to compare the results obtained in this study with those of previous publications involving electrochemical pilot plants applied for petroleum wastewater treatment (Table 6). First of all, it is important to point out that most of the studies did not focus the efficiency on TOG removal, but on COD reduction, taking into consideration that salts, halogenated compounds, metals, and organic matter account for this last methodology, it does not guarantee exclusively a reduction in effluent of the organic compounds, might causing TOG to remain unchanged, which is a major factor for discharge in most countries, since other compounds also contribute to these parameters.

**Table 6:** Summary of the Characteristics, Employed Conditions, and Results of Electrochemical Pilot Plants Applied for Petroleum Wastewater Treatment

electrochemical treatment	operation mode	reactor volume (L)	reaction time (min)/flow rate (L·h^–1^)	anode composition	electrodes’ shape	electrodes’ area (cm^2^)	pH	conductivity (mS·cm^–1^)	current density (A·m^–2^)	wastewater matrix	analysis parameter	initial effluent concentration (mg O_2_·L^1^/mg·L^–1^)	removal (%)	refs
EO + EC	continuous	15	225	Al or Ti/MMO (PtO_2_, IrO_2_, RuO_2_, and Ta_2_O_5_)	plate	5200	7.2	59	25	real oilfield produced water	O&G	152	98	this study
EO	recirculated batch		240	BDD	plate	100	7.0	24	1250	synthetic produced water	TOC	250	75	Tawabini and Basaleh^69^
EO + EC	continuous	600	1500	steel	cylindrical plate	22,500	8.4	3	5	real petroleum refinery effluent	COD	247	50	Hellal, Hamdy, and Abou-Taleb^70^
											O&G	14	50	
											Phenol	3	100	
EO	recirculated batch	22	15	graphite	cylindrical plate	3528	8.0	3	400	real petroleum refinery effluent	COD	199	60	Abou-Taleb et al.^21^
											O&G	29	56	
EO	recirculated batch	-	120	BDD	plate	100	7.0	2	40	real brackish groundwater spiked with phenol	TOC		13	Tawabini et al.^71^
											phenol	50	77	
								36		seawater spiked with phenol	TOC		26	
											phenol	50	100	
EO	recirculated batch	-	15	BDD	plate	100	9.4	45	740	seawater spiked with BTEX^a^	benzene	2	41	Tawabini et al.^72^
											toluene	2	86	
											ethylbenzene	2	100	
											m&*p*-xylene	2	100	
											o-xylene	2	100	
EC	recirculated batch		480	Fe and Al	plate	73,000	8.5	6	9	real petroleum refinery effluent	COD	4689	35	El-Naasa, Surkattib, and Al-Zuhair^68^
											phenol	8	∼0	
											o-cresol	54	∼0	
											m, p-creso	44	∼0	
EO	recirculated batch	4	24	Ti/RuO_2_	perforated plate	660	8.0	-	300	real petroleum refinery effluent	COD	480	84	Ibrahim et al.^73^
EC	recirculated batch	80	31	Fe	plate	1080	7.4	3	3	real oilfield produced water	COD	280	67	Zhao et al.^74^
EO + EC	recirculated batch	-	6	M^b^/C/Fe		10,000	6.5		12	real produced water	COD	5800	90	Ma and Wang^75^

a BTEX abbreviation stands for benzene, toluene, ethylbenzene, and xylene.

b M refers to the active metal.

The application of EC is useful to remove dispersed oil and suspended solids, while dissolved organic pollutants would require the combination of EO + EC. This was evidenced by the tests performed by El-Naasa, Surkattib, and Al-Zuhair (2016),^68^ which presented low efficiency to remove dissolved organic pollutants (phenol, o-cresol, and m,p-cresol) by EC alone but high COD removals. Moreover, EC alone generates high sludge amounts due to the anode abrasion and the floc formation from the coagulant with the suspended solids and dispersed oil.^3^ The disposal of this waste can be extremely challenging and costly, in particular considering offshore operations.^3^

On the other hand, boron-doped diamond (BDD) anodes were the most commonly used anode reported throughout the literature; however, they are not only much more expensive than dimensionally stable anodes (DSA) but also are more susceptible to chloride attack, causing the electrode to become inactivated, requiring more frequent replacement.

It is also important to highlight that all of the other evaluated publications applied higher RT (up to × 120 higher) and/or CD (up to × 50 higher) in the electrochemical reactor to achieve satisfactory performance. Thus, these harsh conditions proposed by other authors might prevent the application in large scale, since (i) large volumes of produced water are generated daily, thus, demanding several units to operate in parallel, demanding high footprint, which might cause it to be an unsuitable solution, (ii) costs considerably increase, which might lead to infeasible energy demands to operate, and (iii) higher RT and CD normally results in an enhancement on the inorganic chlorine subproducts and organochlorines,^14^ possibly causing the acute and chronic toxicities to reach nondisposable levels according to legislations requirements.

Recently, Hellal, Hamdy, and Abou-Taleb (2025)^70^ brought important insights in terms of long-time operation (3 months) under continuous mode, larger-scale applications (600 L reactor and 1500 L·h^–1^ flow rate), and application of low CD (5 A·m^–2^). However, it fails to evaluate the performance of combined EO + EC on high saline wastewaters, which substantially alters the behavior from the brine sample analyzed by the authors. Under high saline media not only the EC mechanism is altered due to a much more aggressive corrosion rate of the sacrificial electrodes and a much more challenging electrical charges stabilization^76^ but also the EO direct at the electrode surface and indirect oxidation reactions on the bulk are intensified, leading to the formation of additional chlorine-based oxidants that might enhance the removal of impurities or lead to the formation of chlorine organic and inorganic subproducts,^76^ needing to be carefully evaluated and optimized.

Hence, the findings from this study demonstrate promising advances toward full-scale industrial electrochemical wastewater treatments for high saline water matrices, such as offshore OPW, due to their high efficiency to remove WSO and TOG, suitable properties for final disposal of the sample, aside from diminished RT, CD, and footprint requirements for operation.

Therefore, taking all of these factors into consideration, it can be concluded that electrochemical units are promising to be applied on an industrial scale, not only in terms of TOG removal efficiency and low final toxicity parameters but also in terms of their size. Although some questions still need further investigation for these units to be implemented effectively and safely, especially when considering offshore OPW treatment.

## 4 Conclusions

Combining the visual results with the performed analyses, the main conclusions about the carried out process are the following: (i) the electrochemical system consists of a hybrid process with EC/electroflotation (destabilizing the molecules’ charges, aggregating the oil and forming flocs, which are floated in the strainer by the gases formed by electro-oxidation in the reactor) and EO (partial oxidation occurs due to short residence time, possibly causing the reduction on the molecules polarity (decarboxylation mechanism), thus leading to the formation of hydrocarbons that easily separate or generating byproducts that are not quantified as TOG); (ii) slight pH change occurs (probably due to reduced residence times in the electrochemical reactor, possibly associated with the H_2_ evolution rate); (iii) the formation of gases occurs (O_2_, Cl_2_, and H_2_ evolutions, being the latter the most likely to occur due to the lower potential required); (iv) the system is slightly pressurized (<7 psi) (this can contribute to greater gas solubilization and higher reaction rates, in addition to helping with phase separation in the strainer); (v) the formation of a second liquid phase occurs at the reactor exit (similar to dispersed oil); vi) at the Unit 1, when applying small RT, the O&G content in the electrochemical reactor outlet is practically the same as that at the entrance (requiring gravitational separation in the strainer to separate the phases); viii) acute and chronic toxicities were reduced or remained the same, suggesting that less hazardous compounds were generated; vii) salinity and solids decreased drastically, probably due to an EC mechanism, getting restrained in the bottom of the strainer, and viii) the scale-up to the pilot plant was successful, and an industrial plant scale-up is promising, in particular in terms of reduced footprint, although further investigations are still required to clarify some aspects such as energy consumption, heat generation, and gas formation.

Taking into consideration the findings from this research, future studies should systematically evaluate operations under different current densities and pH conditions to distinguish real OPW matrices and further evaluate the consistency of the process in terms of TOG removal efficiency, toxicity response, energy demand, heat generation, gas evolution, and byproduct control. This way, the findings can substantiate electrochemical treatments for real offshore OPW, leading to more developed TRLs.
